# A preliminary study through lymphocyte immunophenotyping of the effects of different forms of phosphorus deficiency on dairy cattle blood haematological parameters and immune status

**DOI:** 10.2478/jvetres-2025-0032

**Published:** 2025-06-13

**Authors:** Beata Abramowicz, Łukasz Kurek, Urszula Lisiecka, Katarzyna Żarczyńska

**Affiliations:** 1 Department and Clinic of Animal Internal Diseases, 20-612 Lublin, Poland; 2 Department of Epizootiology and Clinic of Infectious Diseases, University of Life Sciences in Lublin, 20-612 Lublin, Poland; 3 Department of Internal Diseases with Clinic, University of Warmia and Mazury in Olsztyn, 10-719 Olsztyn, Poland

**Keywords:** dairy cattle, lymphocyte immunophenotyping, phosphorus deficiency

## Abstract

**Introduction:**

The study determined the effects of different forms of phosphorus deficiency on white blood cell parameters in cows during the periparturient period and on the cows’ immune status.

**Material and Methods:**

Thirty-two Holstein-Friesian cows aged 3–6 years were divided into four equal groups (I – control group, II – cows with atypical hypophosphataemia, III – cows with post-parturient haemoglobinuria and clinical hypophosphataemia and IV – cows with periparturient recumbency and clinical hypophosphatemia). The experiment used antibodies against CD4^+^ and CD8^+^ T cells (TCD4^+^ and TCD8^+^), CD21^+^ B cells (BCD21^+^) and major histocompatibility complex class II (MHC II). Immunophenotyping was performed by flow cytometry. The morphological examination determined the white and red blood cell and platelet counts, haemoglobin, haematocrit, mean corpuscular volume, mean corpuscular haemoglobin and mean corpuscular haemoglobin concentration. Microscopy of manual smears was performed to ascertain the percentage of leukocytes which was each of the granulocyte, segmented granulocyte and lymphocyte subpopulations.

**Results:**

The TCD4^+^ lymphocyte subpopulation was larger in diseased cows and the largest in group IV animals. The TCD8^+^ lymphocyte subpopulation was smaller in hypophosphataemic Holstein-Friesians, and the smallest in group II. The CD4 : CD8 lymphocyte ratio in all diseased animals was higher than that in the control group, peaking in group II. Expression of MHC II protein was higher in affected than in healthy animals. The BCD21^+^ lymphocyte subpopulation was larger in diseased animals.

**Conclusion:**

Long-term (even minor) phosphorus deficiency in an atypical form affects bovine cellular immunity (CD4 :CD8 ratio). In formerly affected cows, infectious diseases are more likely, even after their inorganic phosphorus level has been restored. Despite the less marked symptoms in animals with atypical hypophosphataemia, such animals have increased susceptibility to infectious agents and greater disturbance in their immune parameters.

## Introduction

Metabolic diseases, especially macronutrient deficiencies, represent a significant health problem in high-yielding dairy cattle. Interest in phosphorus has increased significantly since the Council of the European Community passed a directive concerning the protection of waters against pollution caused by nitrates from agricultural sources (the Nitrates Directive). European Union Member States have a choice of two ways to implement the provisions of the Nitrates Directive, either by designating vulnerable areas or by drawing up an Action Programme (2). The effect of complying with the Directive is also to limit phosphorus content in the soil, which contributes to a decrease in its concentration in feed for ruminants. Research indicates that the amount of phosphorus in the diet of dairy cattle covers the nutritional requirements of these animals and ensures normal reproductive processes and the skeletal system structure. As dairy production increases, the amount of the available element in the diet does not increase in parallel, which can very often lead to a reduction in phosphorus blood concentration (hypophosphataemia) (4).

Phosphorus is involved in numerous biochemical processes, including energy reactions and mineralisation of the skeletal system, and is responsible for normal nerve conduction. Hypophosphataemia is a common problem on high-producing dairy cow farms, especially during the periparturient and early lactation periods. Phosphorus deficiency can occur in clinical, subclinical and atypical forms. The clinical signs of phosphorus deficiency include periparturient recumbency and postparturient haemoglobinuria (PPH). In the initial stage, periparturient recumbency is characterised by difficulty in movement (lameness, soreness and swelling of the joints) and a fluctuating appetite. In the final stage, the animal cannot maintain a standing position, but its appetite is maintained. Post-parturient haemoglobinuria, manifested by anaemia and the presence of haemoglobin in the urine, is often indicative of the presence of hypophosphataemia in the herd. An increasingly common form of low blood inorganic phosphorus (Pi) levels in dairy cattle is the atypical form. The most common symptoms of it include intermittent appetite disorders, a fall in milk production, increasingly pale mucous membranes and lameness. Such symptoms do not clearly indicate a specific cause and often lead to misguided treatment because the underlying disorder is misdiagnosed (7, 11, 15).

During the periparturient period, in addition to phosphorus deficiency, an efficiency loss in the immune system also affects cows, which increases their susceptibility to infectious diseases. The causes of the development of periparturient immunosuppression have not been fully explained and are therefore considered to be a multifactorial process (18). The literature data show that in animals, the phosphorus level has a significant impact on the immune system function (4, 18). Dairy cattle’s granulocyte counts decline concomitantly with (especially chronic) hypophosphataemia, but this does not interfere with phagocytosis activity (4, 5). In addition, it was demonstrated that the phosphorus status in the mammalian body affected the function of both the cellular and humoral immune responses (12, 15). Cellular immunity is associated with T helper cells (CD4^+^) and cytotoxic T cells (CD8^+^), whereas humoral immunity is associated with B cells. The main functions of the aforementioned cells are to recognise antigens that lead to apoptosis of an infected cell and to create an immune memory of the body (9).

The aim of the study was to determine the effects of different forms of phosphorus deficiency on white blood cell parameters in cows during the periparturient period and on their immune status. These effects were investigated through lymphocyte immunophenotyping by flow cytometry.

## Material and Methods

### Animals and group assignment

The study was conducted on 32 Holstein-Friesian cows aged 3–6 years (at an average age of 4.10 years), with a body condition score of 3/5 points. They had been lactating for between 3 and 6 months and giving milk yields of over 9,000 litres. The cows originated from the central Lublin region, Lublin voivodeship. These Holstein-Friesians had been given regular preventive treatments (hoof cleansing and deworming twice a year) but had not been vaccinated against infectious diseases. No infectious diseases occurred in the study during the time the material was collected. The animals were divided into four groups based on their history and clinical examination. Each group comprised eight cows. Group I (control) was composed of clinically healthy animals. In anamnesis, the owner reported normal gestation and delivery and the birth of a healthy calf. Group II included animals whose owners reported a medical history of periodic lack of appetite, lameness and reduced milk production. Periodic lack of appetite was not always manifested during the clinical examination, while lameness and varying degrees of decreased milk yield were observed in all animals. Group III comprised cows with dark red/brown urine, rapidly developing apathy, decreased appetite, a slight decrease in milk yield and pale mucous membranes. In some animals, accelerated heart rates and respiration were noted, and dry faeces were observed in most animals. Based on the clinical signs, a preliminary diagnosis of post-parturient haemoglobinuria was made. Group IV comprised cows with retained appetite and thirst that remained recumbent and were unable to rise. The pelvic limbs of the animals retained superficial and deep sensation, indicating the absence of mechanical damage. The animals exhibited pale mucous membranes and accelerated and heavy breathing.

Observations of animals selected by medical history and clinical examination were carried out over a period of six months leaving the animals unsampled during the period. Based on the information provided by the animal owners and on the authors’ observations, it was found that some animals had suffered paronychia, mastitis, metritis or even respiratory disorders.

The farms used the total mixed ration (TMR) feeding system, and the ration consisted of the same ingredients on each: maize silage, maize grain, hay silage, grass silage, hay, straw, pellets, farm-cultivated cereals, feed additives with a protein content ranging from 18 to 24%, a premix and a mineral and vitamin supplement. Feed rations were developed on the basis of the milk yield, current physiological period and body weight of the cows.

### Blood sampling, biochemical and morphological components, and immunophenotyping of lymphocytes

Blood tests in the control group were conducted according to the principles of dairy herd monitoring carried out at the Department of Animal Internal Diseases of the University of Life Sciences in Lublin, during or after milking and before the first TMR provision (in the morning). Blood samples were collected from diseased animals at the time of the first medical visit, after the clinical examination and before the administration of medications. Blood was collected from the external jugular vein at a volume of 9 mL into tubes without anticoagulant for serum and at 2 mL into tubes containing tripotassium ethylenediaminetetraacetic acid. The Pi concentration was determined in the serum using a Horiba ABX Pentra 400 automatic analyser (Horiba Medical, Montpellier, France). The morphological examination determined the following: white blood cell (leukocyte) count (WBC), red blood cell count (RBC), haemoglobin (HGB), haematocrit (HCT) mean corpuscular volume (MCV), mean corpuscular haemoglobin (MCH), mean corpuscular haemoglobin concentration (MCHC), and the platelet count (PLT), using an automatic Horiba Scil Vet ABC Plus analyser (Horiba Medical). Manual smears were performed on microscope slides; preparations were stained by the May–Grünwald Giemsa method using the Mythic TS haematological smear staining apparatus (Orphée Diagnostics, Geneva, Switzerland), and the leukocyte subpopulation sizes which were neutrophil rod-shaped granulocytes (GP), segmented granulocytes (GS) and lymphocytes (L) were determined as percentages of the total leukocyte population.

Immunophenotyping of lymphocytes was performed using a BD FACSVerse flow cytometer (Becton Dickinson, Franklin Lakes, NJ, USA). The experiment used directly labelled antibodies against cell differentiation antigens: CD4^+^ labelled with Alexa Fluor647 dye, CD8^+^ labelled with fluorescein isothiocyanate (FITC), CD21^+^ labelled with FITC, and major histocompatibility complex class II (MHC II) labelled with R-phycoerythin (RPE) dye. All of the antibodies were sourced from the BIO-RAD company (Hercules, CA, USA). Before conducting the experiments, a titration of the antibodies was carried out to optimise their concentrations. Whole blood samples of a volume of 50 μL were incubated with the addition of 5 μL of the corresponding antibody for 20 min in the dark at room temperature. The samples were then incubated for 20 min at the same temperature with 1 mL of ammonium chloride solution to lyse the red cells in the samples. The final step was to analyse the samples using a flow cytometer. Each test analysed 10,000 leukocytes. Leukocyte subpopulations were gated according to their size and granularity using the forward scatter (FSC) and side scatter (SSC) parameters. The intensity of antibody labelling was analysed using the fluorescence and SSC plots. The results were expressed as a percentage of positive cells within the gated lymphocyte population and as the CD4^+^ : CD8^+^ lymphocyte ratio (the ratio of helper to cytotoxic cells). In order to optimise the gating strategy, appropriate controls were used, and these were analysed under the same conditions as the experimental samples. For the calibration and validation procedures, BD FACSuite CS&T Research Beads (BD Biosciences, San Jose, CA, USA) and BD Calibrite 3 Beads (BD Biosciences) reagents were used. Analyses for each antibody were carried out using the same invariant protocols and instrument settings and at the same applied voltages (233.5 V for FSC, 354.8 V for SSC, 501 V for FITC, 473.1 V for RPE and 544.6 V for Alexa Fluor647). All blood samples were tested at a low flow rate (in “low” mode). The results obtained by the flow cytometry method were confirmed by haematological tests and microscopic evaluation of the leukocytes.

The haematological and biochemical test results were statistically analysed using the Mann–Whitney U test. The calculations were performed with the adoption of P-values < 0.05 and <0.01 as indicators of significant difference.

## Results

**Fig. 1.**
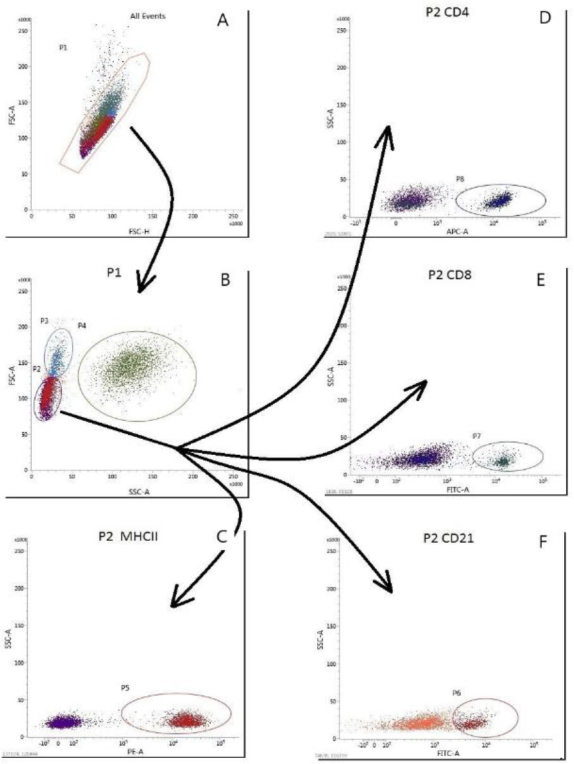
Gating strategy for immunophenotyping of bovine peripheral blood lymphocytes. A – single-cell gating; B – lymphocyte gating; C–F – fluorescence cytograms of positive cells

The flow cytometry gating strategy is presented in Fig. 1. The first step was selecting single cells for analysis on the FSC-A (pulse area) *vs* FSC-H (pulse height) plot (Fig. 1A). Next, the lymphocyte population was gated based on cell size (FSC-A) and granularity (SSC-A) parameters (Fig. 1B). Within the lymphocyte population, the percentage of cells positive for the expression of the analysed antigen was determined on the corresponding fluorescence *vs* SSC-A plots (Fig. 1C–F)

The blood morphology results are presented in Table 1. In all the animal groups under study, a reduction in Pi concentration in relation to the accepted reference standards according to Meyer and Harvey (17) was observed, with the lowest Pi levels noted in animal group III (with PPH) and group IV (with periparturient recumbency).

**Table 1.** Morphology of blood samples of Friesian dairy cows with atypical or clinical hypophosphataemia

Parameters	Scope of standards (17)	Group I (control)	Group II	Group III	Group IV
Pi (mmol/L)	1.5–2.9	1.53 ± 0.10^A^^,^^a^	1.12 ± 0.16^A^^,^^b^	0.37 ± 0.05^B^^,^^c^	0.33 ± 0.15^B^^,^^c^
WBC (×10^9^/L)	6.25–9.5	7.55 ± 1.20^A^^,^^a^	5.63 ± 1.03^B^^,^^b^	4.35 ± 1.45^B^^,^^c^	3.50 ± 1.64^C^^,^^d^
GP (%)	0–2	0.0 ± 0.0	0.0 ± 0.0	0.0 ± 0.0	0.0 ± 0.0
GS (%)	23–37	29.0 ± 7.07^A^^,^^a^	23.0 ± 9.38^A^^,^^b^	37.75 ± 11.09^B^^,^^c^	54.25 ± 7.23^C^^,^^d^
L (%)	53–67	60.0 ± 4.24^A^^,^^b^	68.75 ± 19.26^a^	54.0 ± 15.51b	41.75 ± 5.19c
TCD4+ (%)		24.64 ± 2.04^a^	29.38 ± 3.48^b^	29.44 ± 5.33^b^	32.51 ± 4.27^b^
TCD8+ (%)		12.79 ± 0.57^a^	7.91 ± 0.71^b^	10.84 ± 2.66^A^^,^^b^	11.61 ± 4.27^A^^,^^b^
TCD4/TCD8		1.94 ± 0.23	3.72 ± 0.45	2.77 ± 0.34	2.89 ± 0.57
MHC II (%)		39.29 ± 2.5^a^	46.22 ± 3.83^A^^,^^b^	50.64 ± 11.8^b^	64.20 ± 5.33^c^
BCD21+ (%)		30.56 ± 5.63	34.10 ± 3.83	34.38 ± 5.07	37.17 ± 9.75
RBC (×1012/L)	5.0–7.0	7.03 ± 1.17^a^	6.04 ± 0.59^a^	4.95 ± 0.67^b^	4.91 ± 0.77^b^
HGB (g/L)	105–140	101.5 ± 9.19^A^^,^^a^	90.25 ± 10.72^A^^,^^a^	63.75 ± 22.74bi'	73.75 ± 11.41^B^^,^^b^
HCT (L/L)	0.3–0.4	0.33 ± 0.04^a^	0.28 ± 0.04^A^^,^^b^	0.23 ± 0.03^b^	0.23 ± 0.04^b^
MCV (fL)	40–60	46.5 ± 2.12	47.25 ± 2.22	45.75 ± 2.06	47.25 ± 0.96
MCH (fmol/L)	0.9–1.5	0.97 ± 0.03	0.89 ± 0.03	0.90 ± 0.03	0.93 ± 0.02
MCHC (mmol/L)	16–21	19.44 ± 0.48	19.58 ± 0.27	19.47 ± 0.30	19.70 ± 0.48
PLT (×10^9^/L)	200–800	330.5 ± 17.78	647 ± 269.16	469 ± 114.07	388.5 ± 121.33

1 Pi – inorganic phosphorus; WBC – white blood cell count; GP – granulocytes; GS – segmented granulocytes; L – lymphocytes; TCD4+ – CD4+ T helper cells; TCD8+ – CD8+ T helper cells; MHC II – major histocompatibility complex class II; BCD21+ – CD21+ B cells; RBC – red blood cell count; HGB – haemoglobin; HCT – haematocrit; MCV – mean corpuscular volume; MCH – mean corpuscular haemoglobin; MCHC – mean corpuscular haemoglobin concentration; PLT – platelets;

a, b, c, d – P-value < 0.05;

A, B, C, D – P-value < 0.01

The haematological test showed a reduction in the leukocyte count in all diseased cows. In group IV, the lowest WBC value, neutrophilia (an increase in the percentage of neutrophils) and lymphopaenia (a decrease in the percentage of lymphocytes) were noted. The percentage of leukocytes forming the TCD4^+^ lymphocyte subpopulation was observed to have increased in diseased cows compared to the control group. The highest value was obtained for group IV animals. The percentage which was the TCD8^+^ lymphocyte subpopulation decreased, with the lowest value noted for cows with atypical hypophosphataemia (group II). The CD4: CD8 lymphocyte ratio in all diseased animals was higher than that in the control group, the highest value having been determined in group II. Expression of the MHC II protein in the cows under study was higher than in healthy animals. In addition, it was noted that the BCD21^+^ lymphocyte subpopulation was larger in diseased animals compared to healthy cows. Anaemia was observed in groups II, III and IV. Normocyte hypochromic anaemia was noted in all the animals with atypical phosphorus deficiency, whereas normocytic normochromic anaemia with a more severe course than that in group II was noted in groups III and IV.

## Discussion

Phosphorus supplementation in animal diets may affect the stimulation of peripheral lymphocyte proliferation and function in many species. In a narrative review, Heyer *et al*. (9) showed that in pigs in which the effect of using different levels of phosphorus in the feed on lymphocyte proliferation was examined, there was a positive linear relationship between the amount of phosphorus in the diet and the proliferation of these cells. In our study, we found that phosphorus-deficient cows had a lower percentage of CD8^+^ lymphocytes compared to control animals, and the percentage of CD4^+^ lymphocytes was higher than average. In addition, noted research confirming the results obtained by Engstrom *et al*. (6) that a low-phosphorus diet increased the concentration of 1,25 dihydroxyvitamin D3, which inhibits some lymphocyte functions (including proliferation reactions). It was also observed that the phosphorus supply in feed may affect the B and T lymphocyte counts in the blood (9). Heyer *et al*. (9) incorporated existing assessments of the percentage of T cells by flow cytometry and found that the CD4 : CD8 lymphocyte ratio in broilers fed a phosphorus-rich feed was higher than that in birds with a phosphorus-poor diet. These results contradict our observations in cows, as higher CD4 : CD8 lymphocyte ratios were observed in all phosphorus-deficient groups than in healthy animals. The contradiction may simply reflect species differences, and in addition, the administration of high doses of phosphorus may affect the body differently to the use of normal doses of this element. In cattle studies, the CD4 : CD8 lymphocyte ratio was shown to be >1 (13). However, it can vary depending on the health status of the animal. In cattle with infectious diseases, this ratio was <1. In the present study, in healthy Holstein-Friesian dairy cattle the average CD4 : CD8 ratio was 1.93, whereas Denholm *et al*. (3) demonstrated a 2.38 ratio in the same cow breed from Scotland. In addition, they found that the percentage of T cells and the CD4 : CD8 ratio were largely heritable traits in cattle and could be improved through breeding and manipulation of the animal’s living environment. The CD4 : CD8 lymphocyte ratio is indicative of the functioning of the animal and human immune system. It was observed that decreasing values of this parameter indicated an immune system dysfunction and could be considered an indicator of severe infections and malignant neoplasms (3). An almost twice as high CD4 : CD8 ratio was observed in the present investigation in the atypical form of phosphorus deficiency, than was measured in healthy animals. The authors found no information in the available literature on how phosphorus deficiency may affect the cellular response of the body. Mehrzad and Zhao (16) observed that a CD4 : CD8 ratio of over 4 may be indicative of immune dysregulation in animals. Denholm *et al*. (3) demonstrated that cows with a higher CD4 : CD8 ratio were often prone to developing lameness. In the authors’ own study, the highest CD4 : CD8 ratio was noted in group II animals, in which lameness was also observed at different times. The correlation between immune dysfunction and raised CD4 : CD8 ratios seems to be confirmed by the studies conducted by Denholm *et al*. (3) and Malafaia *et al*. (14) in cattle, in which the consequences of subclinical phosphorus deficiency often included problems associated with reduced animal immunity and increased susceptibility to infectious diseases such as mastitis, metritis, brucellosis and other diseases affecting cow reproduction. In our study, at the time of sample collection, no infectious diseases had been recorded. However, based on the anamnesis and clinical examination carried out at a later stage, it emerged that all animals in group II had suffered disorders such as paronychia, mastitis or metritis, or even respiratory disorders within the six months previous to the identification of the Pi deficiency. In the other groups (III and IV), the percentages of animals with these disease histories were lower and ranged from 40 to 50%. This is consistent with the observations of Denholm *et al*. (3), Malafaia *et al*. (15), and Mehrzad and Zhao (16). However, in the control group, only one cow developed mastitis at a later stage, and one cow developed paronychia.

Our results indicate an immune system dysregulation, as confirmed by a significant increase in the expression of MHC II on lymphocytes in groups III and IV, suggesting immune system activation. This activation may have multiple causes; in this particular case, it may have been the presence of chronic inflammation or immune stress potentially resulting from environmental and dietary factors, as supported by the findings of Jurewicz and Stern (10). Additionally, the observed increase in the expression of the CD21^+^ antigen present on the surface of B lymphocytes in phosphorus-deficient groups may indicate stimulation of the humoral immune response in these animals, which aligns with the observations of Chen *et al*. (1).

## Conclusion

It should be noted that long-term (even minor) phosphorus deficiency in an atypical form clearly affects the cellular immunity of the body and is seen in the CD4: CD8 ratio. In formerly phosphorus-deficient cows, infectious diseases are more likely to be observed, even after the Pi level have been restored. Despite the less marked symptoms in animals with atypical hypophosphataemia, increased susceptibility of cows to infectious agents and greater disturbance in parameters determining immunity are observed. However, the cellular and humoral response of the body to phosphorus deficiency requires further research.
